# The inaugural European emergency medical dispatch conference – a synopsis of proceedings

**DOI:** 10.1186/1757-7241-21-73

**Published:** 2013-09-23

**Authors:** Richard M Lyon, Katarina Bohm, Erika Frischknecht Christensen, Theresa M Olasveengen, Maaret Castrén

**Affiliations:** 1 Emergency Medicine Research Group, Edinburgh, UK; 2 Kent, Surrey & Sussex Air Ambulance Trust, UK; 3 Karolinska Institute, Stockholm, Sweden; 4 Prehospital Emergency Medical Services, Aarhus, Denmark; 5 Ullevål University Hospital, Oslo, Norway; 6 European Resuscitation Council, Edegem, Belgium

## Abstract

The inaugural European Emergency Medical Dispatch conference was held in Stockholm, Sweden, in May 2013. We provide a synopsis of the conference proceedings, highlight key topic areas of emergency medical dispatch and suggest future research priorities.

## Background

Emergency medical dispatch is a vital link in any chain of medical care [1]. Upon receipt of a call requesting medical assistance, the priorities are to characterise the urgency of the reported incident, give medical instructions via telephone and coordinate the dispatch of appropriate medical resources to the correct location. The role of emergency medical dispatch is particularly important in certain conditions, such as out-of-hospital cardiac arrest (OHCA), were the actions of call takers and dispatchers can have a direct effect on clinical outcome of the patient.

The importance of dispatch has not always been routinely recognised [1]. Only recently have elements of dispatch been incorporated into the Utstein recommendations for OHCA [2]. There is a paucity of dispatch research in the published medical literature with many key questions remaining unanswered. Informal discussions have suggested a significant variation in practice with regards to emergency medical dispatch systems across Europe, with the optimum methods of handling calls and dispatching emergency medical resources remaining largely unknown.

Following preliminary discussions at the European Resuscitation Council conference in 2012, there was a perceived need to host a European conference with a specific focus on emergency medical dispatching. The inaugural conference was supported and endorsed by the European Resuscitation Council.

The inaugural European conference on emergency medical dispatching was held on May 14 & 15, 2013 at the Karolinska Institute, Department of Clinical Science and Education, Södersjukhuset, Stockholm, Sweden.

The conference was attended by 54 delegates, representing 8 countries.

The primary aim of the conference was to facilitate introductions and discussions between European emergency medical dispatch leaders, staff, scientists and students to foster initial collaboration. Conference delegates were given the opportunity to compare and contrast different emergency medical dispatch systems, highlight areas of key importance and suggest topics for future dispatch research. We aim to provide a synopsis of the conference proceedings.

### Conference sessions

Conference delegates took part in four facilitated discussion groups on timestamps, protocols, guidelines, identification and symptoms. The summaries of each workshop are incorporated into this conference summary.

### Dispatching systems

The aim of any emergency medical dispatch system is to provide the right resource to the right patient at the right time. The demand placed on prehospital emergency medical resources is increasing throughout Europe. As ambulances become a more scare resource, the need for precise triage and management of the available prehospital resources becomes paramount. There was discussion on the need for emergency medical services to offer alternative care pathways, particularly for patients not requiring immediate transport to hospital or referral to other, non-emergency medical services. Several European systems are introducing non-emergency telephone numbers to offload emergency medical dispatch centres and collaboration on the development of such systems and sharing of experience is likely to be beneficial.

### Call-handling

A variation in how emergency medical calls are handled was highlighted. Some systems employ a single common emergency number, sometimes with a separate number for emergency medical services, whilst others use separate numbers for different emergency services. A key characterisation was whether an emergency call entered the emergency medical dispatch centre directly or was initially routed via an intermediary; either a telecommunications provider or general emergency service centre. Some centres employed a co-listening system with multiple parties listening to a single call, whilst others employed single call takers.

### Guidelines & protocols

There was discussion and comparison of the three main dispatch platforms used in Europe: criteria-based, priority-based and keyword-based. There was a consensus view that all platforms have advantages and disadvantages and further research is warranted to determine the optimum elements of dispatch platforms.

The value of audio review and call transcription of emergency calls was highlighted. It was acknowledged that listening to audio recording is time consuming but is the method of choice for detailed dispatch research. Call transcription and comparison to dispatch protocol allows for individual protocol steps to be analysed in detail and could form the basis of protocol improvement. Listening to audio recordings is not practical on a large scale or for system-wide quality assurance or benchmarking. It was suggested that use of voice recognition software might, in future, facilitate this process.

Analysis of progress through electronic protocols may provide data for this purpose but a validation study is required to compare voice calls to computer algorithm adherence and progression.

The use of key word triggers to assist call-taking was highlighted as an area in need of further research and development.

### Specific conditions

Emergency medical dispatch has an important role to play in determining outcome from immediately life-threatening conditions including out-of-hospital cardiac arrest, major trauma, stroke, acute myocardial infarction, acute respiratory distress, upper airway obstruction and seizures. These conditions, however, make up the minority of calls to emergency medical communication centres, with non-urgent calls representing a large proportion.

### Out-of-hospital cardiac arrest

Dispatch for out-of-hospital cardiac arrest is critical in terms of the need for rapid recognition, early dispatch and provision of telephone CPR advice [3,4]. Several key performance indicators are linked to OHCA call handling and the majority of dispatch research has been conducted in this field. It was felt OHCA should remain as a key condition for any emergency medical communication centre but other conditions should not be overlooked.

Accuracy of cardiac arrest recognition was highlighted as important. Delay in recognition can lead to a delay in telephone CPR instructions and emergency medical services dispatch. OHCA recognition is often confounded by the initial call reporting signs such as collapse, difficulty in breathing, chest pain or seizure. Research is warranted on optimising OHCA recognition rates and it was felt this should be reported as a key performance indicator for any emergency medical dispatch system.

Time to telephone CPR was highlighted as being of key importance and potential causes of delay were discussed [5]. The need to accurately assess breathing, particularly recognition of agonal breathing, airway opening manoeuvres and moving the OHCA patient to a hard surface were all highlighted as causing potential delays to telephone-CPR [6-8]. Several protocols now incorporate steps aimed at identifying agonal respiration and a comparison of these, particularly for sensitivity, has yet to be undertaken.

### Major trauma

Early, accurate dispatch of emergency medical services, particularly advanced trauma or physician-lead teams may have the potential to improve outcome for victims of major trauma [9-11]. Specific telephone instructions on airway maintenance, haemorrhage control and prevention of hypothermia all have the potential to improve care for the major trauma victim. Several centres have developed call-out criteria for physician-lead trauma teams but there is little evidence underpinning these. Some systems prefer to over-triage for major trauma whilst others rely on a scene report from the first emergency medical resources to arrive on scene before dispatching further resources.

Several conference delegates expressed concern that current dispatch protocols do not allow for accurate assessment of the major trauma victim. Little is known about the optimum interrogation strategy for callers reporting accidents and further research is warranted in this area.

### Stroke

Several screening tools (eg FAST) have been developed for the lay person to rapidly identify signs of stroke. Application and piloting of these tools in the dispatch setting may help to improve accuracy and speed of stroke diagnosis [12-14].

It was highlighted that there is limited knowledge on the optimum means of interrogating key specific conditions such as sepsis, although research in this area is currently on-going in Sweden.

### Non-urgent calls

Non-urgent calls represent a large proportion of ambulance service workload [15]. Increasingly, patients with chronic conditions are putting strain on dispatch systems. Several systems now employ physicians within the emergency medical dispatch centre to provide medical advice on the triage and management of such cases. A Danish study is currently underway aiming to characterise the nature and epidemiology of all calls to an emergency medical dispatch centre.

### Personnel

The qualifications and skill mix of call handlers and dispatchers were discussed in detail. Scandinavian systems, except Finland, frequently employ nurses as calls takers with paramedics being used in a dispatch role. In the United Kingdom, both call takers and dispatchers have minimal medical training. The optimum qualification and staffing of emergency medical communication centres remains a key area for comparison and investigation.

### Call taker and dispatcher training

There is great variation in call taker and dispatcher training across Europe with training periods ranging from several weeks to nearly 2 years. Dispatch personnel encounter variable numbers of calls per year and varying proportions of high-acuity calls across services. Previous research has shown that outcome from OHCA can be influenced by the experience of call taker handling the initial emergency call [16].

The use of experimental scenarios and simulation training may be of benefit in training both call takers and dispatchers. Translation into different languages would allow protocol and system comparison across countries.

### Data

The storage, handling and analysis of dispatch data was discussed in detail. It was agreed emergency medical dispatch was a data-rich environment with the potential for valuable future research. Some European prehospital emergency medical systems have developed integrated dispatch and ambulance data systems that are populated in real-time using tablet technology. Even in the presence of the Utstein dispatch criteria, it was agreed a standardised minimum dispatch data set is needed to allow comparison between dispatch systems [2,17]. Accurate linkage of dispatch, prehospital and hospital data sets is required to allow outcome analysis and to evaluate dispatch accuracy.

The legal definition of the caller and dispatcher patient record appeared to vary greatly amongst the represented countries. In some countries the legal record commences in the prehospital setting, whilst in other it does not commence until the patient reaches hospital.

### Quality assurance

There was consensus agreement that quality assurance is an integral part of any emergency medical dispatch system. Most quality assurance focuses on time targets. Time to answer the emergency call, time to dispatch and time to arrival on-scene are routinely measured but the precise definition of each time point is variable and consistency is required.

Many emergency dispatch centres collect some time stamp data as recommended in the Utstein criteria but these are variable [2].

It was agreed non-automated quality assurance indicators, such as time or accuracy of cardiac arrest diagnosis or time to telephone-assisted CPR were important. Such variables have been shown to influence survival from OHCA but are not routinely measured or reported. Precise definitions and novel data interrogation systems are needed to allow quality assurance on a system-wide basis and allow comparison between systems.

### Future research

Several potential areas of future dispatch research were discussed. The role of telemedicine, in particular the use of mobile telephone technology to transmit real time photos and video from the scene was discussed. Mobile technology now exists that also allows for transmission of physiological data, including electrocardiography, which may facilitate more accurate dispatch. Following a presentation from Edinburgh, Scotland, the role of behavioural psychology and language analysis could provide a platform for future work on dispatch protocols and call taker behaviour.

Further research is warranted on automated dispatch systems to facilitate call prioritisation and allow rapid dispatch.

## Conclusion

The inaugural European emergency medical dispatch meeting proved to be successful and positive feedback was received from all delegates. Variation in emergency medical dispatch was highlighted, as was the need to collaborate internationally in order to undertake future dispatch research.
